# Increased biosynthesis and accumulation of cholesterol in maternal plasma, but not amniotic fluid in pre-eclampsia

**DOI:** 10.1038/s41598-018-37757-3

**Published:** 2019-02-07

**Authors:** Seung Mi Lee, Ju-Yeon Moon, Byeong-Yun Lim, Sun Min Kim, Chan-Wook Park, Byoung Jae Kim, Jong Kwan Jun, Errol R. Norwitz, Man Ho Choi, Joong Shin Park

**Affiliations:** 1 Department of Obstetrics & Gynecology, Seoul National University College of Medicine, Seoul, Korea; 2 College of Pharmacy, The Catholic University of Korea, Gyeonggi-do, Korea; 3 Molecular Recognition Research Center, Korea Institute of Science & Technology, Seoul, Korea; 4 Department of Obstetrics & Gynecology, Seoul Metropolitan Government Seoul National University Boramae Medical Center, Seoul, Korea; 5 Department of Obstetrics & Gynecology, Tufts University School of Medicine, Boston, MA USA

## Abstract

Preeclampsia is one of the most serious complications during pregnancy, defined as development of hypertension during late pregnancy affecting other organ systems (proteinuria, thrombocytopenia, renal insufficiency, liver involvement, cerebral symptoms or pulmonary edema). Preeclampsia is known to be associated with significant dyslipidemia, but the cause or mechanism of this metabolic aberration is not clear. Quantitative analysis of cholesterol precursors and metabolites can reveal metabolic signatures of cholesterol, and provide insight into cholesterol biosynthetic and degradation pathways. We undertook this study to compare the metabolic signatures of cholesterol in serum and amniotic fluid collected from women who delivered in the late preterm period. Matching serum and amniotic fluid samples were collected from women who delivered in the late preterm period (34-0/7–36-6/7 weeks), had undergone amniocentesis within 3 days of delivery, had no evidence of rupture of membranes or intra-amniotic infection/inflammation, and who had not received antenatal corticosteroid prior to amniocentesis. Patients were classified into 3 groups according to the etiology of their preterm birth: Group 1, preeclampsia; Group 2, spontaneous preterm labor; Group 3, other maternal medical indications for iatrogenic preterm birth. Quantitative metabolite profiling of cholesterols was performed using gas chromatography-mass spectrometry. A total of 39 women were included in the analysis (n = 14 in Group 1, n = 16 in Group 2, n = 9 in Group 3). In maternal blood, patients in Group 1 had significantly higher ratios of cholesterol/desmosterol and cholesterol/7-dehydrocholesterol (which represent 24- and 7-reductase enzyme activity, respectively) than those in Group 3 (p < 0.05 for each), which suggests increased cholesterol biosynthesis. In contrast, patients in Group 1 had significantly decreased ratios of individual cholesterol esters/cholesterol and total cholesterol esters/cholesterol than those in Groups 3 (p < 0.01 for each), suggesting increased reverse cholesterol transport. No differences in cholesterol ratios were found in amniotic fluid among the 3 groups. In conclusion, the metabolic signatures of cholesterol suggest increased cholesterol biosynthesis and accumulation in the maternal blood (but not amniotic fluid) of women with preeclampsia.

## Introduction

Preeclampsia is a multisystem disorder characterized by maternal hypertension and end-organ damage, complicating 3–4% of pregnancies^1,2^. About 10–16% of all maternal deaths are related to preeclampsia^3–5^. Abnormal trophoblast invasion and endothelial dysfunction is the main participants in the pathogenesis of preeclampsia. In placentas of preeclamptic women, endothelial damage and lipid accumulation in myointimal cells and macrophages, which is atherosis, is more common finding, resulting in abnormally narrow spiral arterioles and subsequent impaired placental perfusion^6^.

The increased level of circulating lipids may result in accelerated accumulation within endothelial cell. In previous reports, preeclamptic patients showed higher serum levels of triglycerides, total cholesterol and low-density lipoprotein cholesterol (LDL-C) than normal pregnant women^7,8^, whereas the relationship between dyslipidemia and preeclampsia was not consistent in other studies^9,10^. In addition to lipoprotein concentrations, the levels of endogenous and exogenous sterols reflect cholesterol metabolism, including biosynthesis (cholesterol precursors), degradation (hydroxycholesterols) and absorption (plant sterols)^11^. In our previous study, the association between cholesterols and lipid profiles was observed^12^, and understanding the mechanisms underlying cholesterol metabolism may implicate pathophysiology and therapeutic response to dyslipidemias and metabolic syndrome^13,14^.

Due to quantitative analysis of cholesterol precursors and metabolites provides insight into metabolic signatures of cholesterol, we evaluated the metabolic profiling in both maternal plasma and amniotic fluid (AF) obtained from pregnant women who delivered in the late preterm period because of preeclampsia and compared these results to that measured in women who delivered at a comparable gestational age due to spontaneous preterm labor or a maternal medical indication. We evaluated whether metabolic signature suggesting cholesterol biosynthesis and accumulation pathway be altered in women with preeclampsia.

## Materials and Methods

### Study design

In this retrospective cohort study, consecutive singleton pregnant women who delivered in the late preterm period (34-0/7–36-6/7 weeks) and who met the following criteria were included: (1) clinically-indicated amniocentesis within 3 days of delivery; (2) no evidence of rupture of membranes; (3) no evidence of intra-amniotic infection/inflammation in the AF; and (4) no antenatal corticosteroids prior to amniocentesis. Cases with twin pregnancy, a major fetal structural anomaly or aneuploidy, or intrauterine fetal death were excluded. This study was approved by the Institutional Review Board of the Seoul National University Hospital (Approval Number 1606-051-770), and patients provided written informed consent for the sample collections and use of biologic materials for research purposes. And all experiments were performed in accordance with relevant guidelines and regulations.

### Etiology of late preterm birth

Patients were classified into 3 groups according to the etiology of their preterm birth: Group 1, preeclampsia; Group 2, spontaneous preterm labor; Group 3, other maternal medical indications for iatrogenic preterm birth. Preeclampsia and the severity of preeclampsia were diagnosed using standardized criteria suggested by the American College of Obstetricians and Gynecologists^1^. Briefly, preeclampsia was defined as hypertension during late pregnancy and evidence of multiorgan involvement including proteinuria, thrombocytopenia, renal dysfunction, liver involvement, central nervous system perturbations, or pulmonary edema.

### Sample collection and preparation

Amniocentesis was done prior to preterm delivery by transabdominal amniocentesis or at the time of cesarean delivery (by inserting a needle into the fetal membranes and withdrawing fluid under direct visualization)^15^. In all cases, the amniocentesis was clinically indicated to determine the presence or absence of intra-amniotic infection/inflammation or to document fetal lung maturity. Amniotic fluid was cultured for aerobic and anaerobic bacteria as well as for genital mycoplasmas, and was examined in the hemocytometer chamber to determine the white blood cell (WBC) count. Cases with positive AF culture (intra-amniotic infection) or with intra-amniotic inflammation (defined as an AF WBC ≥19/mm^3^ as previously reported^16,17^), were excluded from the analysis. At the time of amniocentesis, maternal venous blood samples were obtained, and collected into tubes containing EDTA. The AF and maternal plasma samples were centrifuged at 2,000 rpm for 10 min and stored at −70 °C until assayed.

### Chemicals

All reference standards were obtained from Steraloids (Newport, RI, USA). Other chemicals included the deuterium-labeled internal standards (ISs), 2,2,3,4,4,6-*d*_6_-cholesterol and 2,2,3,4,4,6-*d*_6_-cholesteryl stearate (C/D/N Isotopes, Pointe-Claire, Quebec, Canada) for cholesterol, plant sterols and cholesteryl esters (CEs), respectively, and 25,26,26,26,27,27,27-*d*_7_-4β-hydroxycholesterol and 25,26,26,26,27,27,27-*d*_7_-27-hydroxycholesterol (Avanti Polar Lipids, Alabaster, AL, USA) for 3 cholesterol precursors and 8 hydroxycholesterols (OHCs). For trimethylsilylation (TMS), *N*-methyl-*N*-trifluorotrimethylsilyl acetamide (MSTFA), ammonium iodide (NH_4_I), and dithioerythritol (DTE) were purchased from Sigma (St. Louis, MO, USA). The hybrid SPE-precipitation cartridge (H-PPT, 1 mL, 30 mg) was supplied by Supelco (Bellefonte, PA, USA). All organic solvents were of analytical and HPLC grades and were purchased from Burdick & Jackson (Muskegon, MI, USA).

### Metabolic signatures of cholesterol

Quantitative metabolite profiling of cholesterols was performed using gas chromatography-mass spectrometry as previously reported^18,19^. Briefly, samples of serum (20 μL) and AF (50 μL) were spiked with 20 μL of the IS mixtures (*d*_6_-cholesterol and *d*_6_-cholesteryl stearate; 100 μg/mL, *d*_7_-4β-hydroxycholesterol and *d*_7_-27-hydroxycholesterol; 20 ng/mL) and added to 0.5 mL methanol. The mixture was vortexed for 5 min and centrifuged for 2 min at 12,000 rpm for protein precipitation. Samples were then loaded into H-PPT cartridges and eluted 3 times with 0.5 mL of methanol. The combined eluate was evaporated under an N_2_ evaporator at 40 °C and dried in a vacuum desiccator over P_2_O_5_/KOH for 30 min. The dried residues were derivatized with 40 μL of MSTFA/NH_4_I/DTE (500:4:2, v/w/w) for 20 min at 60 °C, and 2 μL of the resulting mixture was injected into the GC-MS system.

### GC-MS analysis

GC-MS was performed with an Agilent 6890 Plus gas chromatograph interfaced with a single-quadrupole Agilent 5975C MSD (Agilent Technologies; Palo Alto, CA, USA). Each sample (2 μL) was injected in split mode (10:1) at 300 °C and separated through a MXT-5 capillary column (15 m × 0.25 mm I.D., 0.25 μm film thickness, Silcosteel-treated stainless steel; Restek; Bellefonte, PA, USA). The oven temperature was initially held at 265 °C for 5 min, then increased to 280 °C at a rate of 2 °C/min, and finally increased to 380 °C at 5 °C/min (held for 3 min). The carrier gas was ultra-high-purity helium at a column head pressure of 89.6 kPa (13 psi; column flow: 1.1 mL/min at an oven temperature of 265 °C).

### Statistical analysis

The calibration curve consisted of a blank sample (matrix sample processed without internal standards), a zero sample (matrix sample processed with internal standards), and 11 calibrators from LOQ to the expected range in the sample. Good linear regression results in a two-curves calibration standard per concentration level were achieved for all compounds analyzed with the higher correlation coefficient (r^2^) than 0.99. Quantitative results for individual sterols and their metabolic ratios, which were calculated by dividing the concentration of the substrate by the concentration of its metabolite (an indicator of enzyme activity), are expressed as means ± standard deviation (SD). Continuous data were analyzed using the Mann-Whitney U test or Kruskal-Wallis test, and categorical data were analyzed with Fisher’s exact test or Chi-square test as appropriate. Statistical analyses were conducted using the IBM SPSS version 20. *P* < 0.05 was considered statistically significant.

## Results

### Study population

A total of 39 women met the inclusion criteria and were included in the final analysis; 14 with preeclampsia (Group 1), 16 with spontaneous preterm labor (Group 2), and 9 with other maternal medical indications for iatrogenic preterm birth (Group 3). In Group 1, 11 cases were diagnosed with severe preeclampsia and 3 with non-severe preeclampsia, according to the ACOG standardized criteria^1^. In Group 3, the indications for iatrogenic preterm birth were critical maternal medical disease (n = 4), maternal physical discomfort (n = 2, osteogenesis imperfecta in 1 case and intractable back pain in 1 case), poorly controlled maternal depression (n = 1), and maternal request for late preterm delivery due to history of prior intrauterine fetal death after confirmation of fetal lung maturity (n = 2).

Table 1 shows the clinical characteristics of the study population according to the etiology of the late preterm birth. Maternal age, parity, and gestational age at delivery were not significantly different among the three groups. However, the fetuses of women with preeclampsia (Group 1) had significantly lower birthweight than those in Groups 2 and 3.

**Table 1:** Characteristics of the study population according to the etiology of the late preterm birth.

	Group 1	P^a^	Group 2	P^b^	Group 3	P^c^	P^d^
	Preeclampsia (n = 14)		Preterm labor (n = 16)		Other maternal-fetal indication (n = 9)		
Maternal age (years)	30.9 ± 4.1	0.498	32.3 ± 3.8	0.637	32.4 ± 2.8	0.277	0.527
Nulliparity	7 (50%)	0.457	5 (31%)	0.364	1 (11%)	0.086	0.151
Pre-gestational body-mass index (kg/m^2^)	21.9 ± 3.2 (n = 9)	0.552	20.5 ± 3.1 (n = 11)	0.062	24.5 ± 4.0 (n = 6)	0.272	0.167
Gestational age at amniocentesis (weeks)	35.7 ± 1.1	0.984	35.7 ± 0.8	0.677	35.9 ± 0.8	0.688	0.882
Gestational age at delivery (weeks)	35.7 ± 1.1	0.984	35.7 ± 0.8	0.760	35.9 ± 0.7	0.781	0.940
Birth weight (g)	2278 ± 586	0.070	2634 ± 472	0.301	2798 ± 290	**0.013**	**0.031**
Small for gestational age	2 (14%)	0.586	1 (6%)	1.000	0 (0%)	0.502	0.437
Sex (male)	7 (50%)	1.000	8 (50%)	0.677	3 (33%)	0.669	0.679
Diabetes during pregnancy	0 (0%)	1.000	1 (6%)	1.000	1 (11%)	0.391	0.482
Cesarean delivery	14 (100%)	0.103	12 (75%)	0.260	9 (100%)	(−)	**0.041**

*All values are given as mean ± standard deviation.

P^a^: Comparison between groups 1 and 2.

P^b^: Comparison between groups 2 and 3.

P^c^: Comparison between groups 1 and 3 P^d^: Comparison among groups 1, 2 and 3.

### Cholesterol signatures in maternal blood

Patients in Group 1 had significantly higher ratios of cholesterol/desmosterol and cholesterol/7-dehydrocholesterol, representing 24- and 7-reductase enzyme activity, respectively, than those in Group 3 (p < 0.05 for each, Table 2). This suggests increased cholesterol biosynthesis. In contrast, patients in Group 1 had the lower ratios of individual cholesterol esters/cholesterol and total cholesterol esters/cholesterol than those in Group 3 (p < 0.01 for each), suggesting increased reverse cholesterol transport.

**Table 2:** Metabolite profiling of cholesterols in maternal serum samples.

	Group 1 Preeclampsia (n = 14)		Group 2 Preterm labor (n = 16)		Group 3 Other maternal-fetal indication (n = 9)		K-W test	Mann Whitney U test		
	mean	SD	mean	SD	mean	SD	p-value	Group 1 vs. 2	Group 1 vs. 3	Group 2 vs. 3
***Concentration (μg/mL)***										
Cholesterol	588.4	215.7	599.3	165.0	529.7	117.9	0.686	0.759	0.734	0.357
Cholesterol laurate	1.8	0.4	1.9	0.5	2.8	3.2	0.937	0.886	0.926	0.677
Cholesterol myristate	52.5	40.2	54.1	25.8	45.3	20.9	0.549	0.313	0.926	0.487
Cholesterol palmitate	736.8	534.3	627.2	355.8	509.8	184.0	0.852	0.951	0.557	0.718
Cholesterol oleate, linoleate & stearate	1460.9	558.0	1587.0	442.6	1571.2	443.7	0.757	0.580	0.516	0.934
Cholesterol arachidonate	371.5	229.0	383.0	172.3	341.1	118.5	0.787	0.525	0.688	0.890
∑Chol-O, Li, S, A	1832.4	749.4	1970.0	526.3	1912.2	528.9	0.863	0.608	0.781	0.934
∑Cholesterol esters	2623.5	1255.1	2653.2	726.5	2470.1	650.8	0.830	0.498	0.975	0.890
Sitosterol	3.1	1.7	3.5	0.8	3.2	0.6	0.272	0.154	0.600	0.276
Campesterol	2.8	1.1	2.8	0.8	2.8	1.2	0.758	0.580	0.734	0.522
Stigmasterol	1.4	0.6	1.4	0.1	1.4	0.0	0.177	0.257	0.250	0.890
***Concentration (ng/mL)***										
Desmosterol	145.6	48.5	177.3	74.8	172.8	62.1	0.293	0.166	0.224	1.000
7-Dehydrocholesterol	170.6	58.5	201.1	79.5	203.4	57.1	0.316	0.257	0.179	0.718
Lathosterol	962.8	764.3	1005.7	524.9	883.1	266.7	0.796	0.580	0.734	0.677
Lanosterol	89.9	31.9	102.8	33.8	104.6	45.0	0.344	0.166	0.369	0.718
7α-OHC	235.2	132.9	266.3	242.6	197.8	86.9	0.843	0.822	0.516	0.846
7β-OHC	163.9	72.1	179.3	136.6	140.6	46.5	0.799	0.759	0.403	0.978
4β-OHC	29.4	9.7	32.7	12.1	27.5	7.5	0.642	0.667	0.600	0.388
27-OHC	28.5	8.1	28.2	7.1	25.1	4.7	0.753	0.918	0.516	0.522
24-OHC	9.3	7.2	9.3	5.7	6.3	2.7	0.516	0.400	0.781	0.329
***Metabolic ratios***										
**Chol/Desmo**	**4.032**	**0.689**	**3.546**	**0.629**	**3.273**	**0.974**	0.061	0.058	**0.039**	0.637
**Chol/7-DHC**	**3.440**	**0.532**	**3.093**	**0.508**	**2.714**	**0.740**	**0.028**	0.058	**0.019**	0.207
7-DHC**/**Latho	0.252	0.180	0.240	0.128	0.242	0.075	0.687	1.000	0.477	0.452
Latho**/**Lano	9.712	4.205	9.443	3.286	8.965	2.383	0.940	0.918	0.688	0.934
Desmo**/**Lano	1.679	0.493	1.727	0.447	1.810	0.755	0.773	0.580	0.600	0.760
Chol**/**Lano	6.693	1.933	5.988	1.222	5.560	1.969	0.271	0.355	0.159	0.329
Sito*1000**/**Chol	5.755	1.395	6.152	1.855	6.242	1.210	0.755	0.759	0.477	0.677
Campe*1000**/**Chol	4.822	1.137	4.913	1.537	5.218	1.215	0.726	0.984	0.439	0.559
Stigma*1000/ Chol	2.653	0.941	2.542	0.733	2.761	0.760	0.691	0.918	0.643	0.357
Campe/ Sito	0.853	0.165	0.813	0.183	0.846	0.177	0.755	0.448	0.975	0.718
Stigma/ Sito	0.453	0.074	0.419	0.055	0.441	0.061	0.249	0.154	0.643	0.207
7α-OHC/ Chol	0.398	0.188	0.453	0.375	0.358	0.119	0.852	0.697	0.600	0.803
7β-OHC/ Chol	0.284	0.096	0.302	0.192	0.260	0.040	0.876	0.728	0.688	0.718
7α-OHC/ 7β-OHC	1.344	0.330	1.328	0.409	1.344	0.308	0.943	0.728	0.975	0.890
4β-OHC/ Chol	0.051	0.011	0.055	0.014	0.052	0.006	0.491	0.313	0.734	0.388
27-OHC/ Chol	0.051	0.011	0.050	0.019	0.048	0.007	0.438	0.294	0.926	0.301
24-OHC/Chol	0.014	0.007	0.015	0.006	0.012	0.005	0.413	0.608	0.477	0.187
Chol-L**/**Chol	0.003	0.001	0.003	0.001	0.005	0.005	0.566	0.951	0.403	0.329
Chol-M**/**Chol	0.082	0.040	0.090	0.036	0.083	0.028	0.890	0.637	0.926	0.846
Chol-P**/**Chol	1.205	0.537	1.060	0.495	0.991	0.363	0.572	0.552	0.250	0.890
**Chol-O, Li & S/Chol**	**2.472**	**0.244**	**2.693**	**0.467**	**2.932**	**0.337**	**0.022**	0.093	**0.005**	0.229
Chol-A**/**Chol	0.609	0.213	0.635	0.207	0.631	0.173	0.847	0.728	0.557	0.978
**∑Chol-O, Li, S, A/Chol**	**3.081**	**0.344**	**3.328**	**0.469**	**3.563**	**0.417**	**0.020**	0.085	**0.007**	0.169
∑Cholesterol esters/Chol	4.371	0.801	4.482	0.751	4.642	0.616	0.579	0.697	0.336	0.487

NS, not significant.

In the study population, there are 2 women with diabetes. The altered metabolic signature (higher ratios of cholesterol/desmosterol and cholesterol/7-dehydrocholesterol, and lower ratios of individual cholesterol esters/cholesterol and total cholesterol esters/cholesterol in Group 1 than those in Group 3) remained significant, even after excluding 2 cases with diabetes.

### Metabolite profiling in amniotic fluid

No differences in metabolite profiling of cholesterol were found in AF among the 3 groups (Table 3).

**Table 3:** Metabolite profiling of cholesterols in amniotic fluid.

	Group 1 Preeclampsia (n = 14)		Group 2 Preterm labor (n = 16)		Group 3 Other maternal-fetal indication (n = 9)		K-W test
	mean	SD	mean	SD	mean	SD	p-value
***Concentration (μg/mL)***							
Cholesterol	2.4	0.7	3.8	5.5	2.1	0.5	0.785
Cholesterol laurate	0.4	0.0	0.4	0.0	0.4	0.0	1.000
Cholesterol myristate	0.7	0.1	0.7	0.1	0.7	0.0	0.454
Cholesterol palmitate	1.8	0.4	1.7	0.6	1.7	0.3	0.627
Cholesterol oleate, linoleate & stearate	2.0	0.4	1.8	0.6	2.1	0.4	0.286
Cholesterol arachidonate	ND^a^	—	ND	—	ND	—	NA^b^
∑Cholesterol esters	4.5	1.0	4.4	1.3	4.6	0.6	0.508
Sitosterol	0.2	0.03	0.2	0.0	0.2	0.1	0.373
Campesterol	ND	—	0.2	0.0	0.2	0.0	1.000
Stigmasterol	0.2	0.0	0.2	0.0	0.2	0.0	1.000
***Concentration (ng/mL)***							
Desmosterol	31.0	7.7	32.8	10.0	29.9	4.6	0.768
7-Dehydrocholesterol	21.6	1.3	23.2	5.5	21.9	1.2	0.492
Lathosterol	71.1	36.7	93.8	90.2	50.2	5.8	0.770
Lanosterol	22.6	12.7	22.4	16.2	13.6	3.2	0.500
7α-OHC	3.5	1.6	3.5	2.1	3.2	2.5	0.760
7β-OHC	2.5	1.0	2.8	1.2	2.4	1.1	0.714
4β-OHC	ND	—	ND	—	ND	—	NA
27-OHC	ND	—	ND	—	ND	—	NA
24-OHC	ND	—	ND	—	ND	—	NA
***Metabolic ratios***							
Chol**/**Desmo	0.081	0.033	0.098	0.090	0.073	0.027	0.825
Chol**/**7-DHC	0.110	0.032	0.137	0.129	0.097	0.027	0.745
7-DHC**/**Latho	0.365	0.144	0.352	0.156	0.441	0.051	0.502
Latho**/**Lano	3.618	0.895	4.271	1.520	3.851	0.878	0.223
Desmo**/**Lano	1.740	0.917	2.032	1.125	2.310	0.741	0.477
Chol**/**Lano	0.135	0.054	0.149	0.083	0.162	0.054	0.594
Sito*1000**/**Chol	87.604	29.851	103.467	51.266	113.306	38.767	0.330
Campe*1000**/**Chol	NA	—	69.892	55.706	90.909	—	0.655
Stigma*1000/ Chol	69.048	3.367	88.551	51.381	78.788	17.142	0.865
Campe/ Sito	NA	—	0.889	0.192	0.500	—	0.157
Stigma/ Sito	1.000	0.000	0.917	0.154	0.750	0.354	0.431
7α-OHC/ Chol	1.619	0.985	1.720	1.499	1.570	1.150	0.850
7β-OHC/ Chol	1.130	0.545	1.343	0.998	1.199	0.545	0.849
7α-OHC/ 7β-OHC	1.386	0.270	1.186	0.402	1.176	0.406	0.302
Chol-L**/**Chol	0.160	0.000	0.189	0.139	0.192	0.082	0.975
Chol-M**/**Chol	0.305	0.075	0.340	0.199	0.334	0.065	0.793
Chol-P**/**Chol	0.791	0.214	0.832	0.528	0.826	0.149	0.905
Chol-O, LI, S**/**Chol	0.886	0.285	0.904	0.583	1.003	0.153	0.492
∑Cholesterol esters**/**Chol	2.002	0.575	2.154	1.343	2.219	0.343	0.718

^a^ND, not detected.

^b^NA, not applicable.

## Discussion

### Novel findings of this study

(1) In maternal serum, patients with preeclampsia had higher ratios of cholesterol/desmosterol and cholesterol/7-dehydrocholesterol (which represent 24- and 7-reductase enzyme activity, respectively) than those without preeclampsia, suggesting increased cholesterol biosynthesis; (2) In contrast, patients with preeclampsia had decreased ratios of individual cholesterol esters/cholesterol and total cholesterol esters/cholesterol than those without preeclampsia, suggesting increased reverse cholesterol transport; (3) no differences were found in cholesterol measurements and ratios in AF between patients with and without preeclampsia.

### Interpretation of the result of the current study

Although the association between preeclampsia and dyslipidemia has been previously described and is biologically plausible, it has not been a consistent finding in the literature^7–9,20,21^. In the largest report, a 2014 meta-analysis incorporating 74 studies, preeclampsia was associated with dyslipidemia including elevated total cholesterol, non-HDL (high density lipoprotein) cholesterol, and triglyceride levels during all trimesters of pregnancy^10^. However, other investigators could not find association between preeclampsia and lipid and/or lipoprotein levels^22,23^.

In the current study, cholesterol biosynthesis was increased and reverse cholesterol transport was also increased in women with preeclampsia. However, levels of free (biologically active) cholesterol in the maternal circulation were not different among the three groups of cases. This contrast result may be explained by two points of view. First, this negative result in the level of cholesterol may be originated from the small number of cases. Second, these findings suggest that metabolic profiling of cholesterol might detect subtle changes in cholesterol metabolism, and that such changes may be evident even before the onset of definite changes in cholesterol concentration in maternal blood. Indeed several other studies did not find significant correlation between cholesterol levels and preeclampsia^22,23^.

### Possible mechanism of altered cholesterol metabolism

Several mechanisms can be proposed to explain the association between cholesterol metabolic change and preeclampsia. First, dyslipidemia may result from excessive oxidative stress leading to generalized endothelial dysfunction, which is the pathogenic hallmark of preeclampsia^24^. Moreover, accumulation of lipid within the endothelial cell lining of the maternal spiral arteries may impair normal trophoblast invasion, possibly by altering prostaglandin production^25,26^. Second, both dyslipidemia and preeclampsia are associated with the development of metabolic syndrome and related vascular disorders^27^. Lastly, obesity may be the linking mechanism between dyslipidemia and preeclampsia. The biochemical markers of obesity were altered in women with preeclampsia, suggesting sharing mechanism among obesity, preeclampsia, and long-term cardiovascular disease^28^.

Regarding the risk of preeclampsia, several recent studies have reported the changes in cholesterol homeostasis and metabolism. HDL cholesterol efflux capacity (the ability of HDL to remove cholesterol from macrophages) has been reported as a new biomarker for cardiovascular risk, and also as a biomarker for preeclampsia^29,30^. Accelerated lipid metabolism outbalancing remnant removal mechanisms has been suggested as the contributor to the endothelial dysfunction in preeclampsia^31^. The relationship between altered cholesterol homeostasis/ metabolism and preeclampsia should be evaluated in further studies.

### The strength and limitation of the current study

Management guidelines published by the Society for Maternal-Fetal Medicine (SMFM) in 2011 recommend that women with severe preeclampsia (without an absolute indication for imminent delivery) be delivered at or after 34 weeks of gestation^32^. As such, most cases of severe preeclampsia are delivered in the late preterm period. To fully understand the pathophysiologic changes in biologic samples collected from women with preeclampsia, appropriate control samples are needed for comparison; however, most healthy pregnant women are delivered at term. Most studies that have examined the pathophysiologic changes in biomaterials such as AF or placenta collected from women with preeclampsia have designated women who delivered preterm (after spontaneous preterm labor or preterm premature rupture of membranes) as the most appropriate controls. However, this may result in difficulty in interpreting the results, because differences could be explained by the presence of preeclampsia or by the pathologic mechanism that led to the spontaneous preterm parturition. To minimize this issue, we included as controls for preeclampsia not only women who deliver because of spontaneous preterm labor, but also women who delivered in the late preterm period because of maternal medical indications. In the current study, we did not evaluate women who delivered term neonates. They would have been true controls, but they would have not been matched for gestational age, also resulting in difficulty in the interpretation of the result when comparing with preeclamptic women who ended up in preterm birth.

Preeclampsia is known as heterogeneous disease with various clinical spectrums. In the current study, we included only preeclamptic women who delivered at late preterm period. Therefore women with early onset preeclampsia with severe feature that needed urgent delivery before 34 weeks of gestation were not included. In addition, the frequency of small for gestational age was 14.3%, suggesting that the primary feature of preeclampsia in the current study is mainly maternal condition rather than fetal growth impairment. More studies are needed to evaluate the cholesterol metabolism and various features of preeclampsia.

In addition, there were 14 cases with preeclampsia, including 11 cases with severe preeclampsia and 3 cases with non-severe preeclampsia. In terms of severity of preeclampsia, the changes of metabolic profiling were more pronounced in women with severe versus non-severe preeclampsia (data not shown), although there is not really enough power to make any firm conclusions from this because of small number of cases with non-severe preeclampsia.

Although the present retrospective study was conducted with plasma and amniotic fluid obtained from pregnant women, who were delivered between 1999 and 2007, most steroids including cholesterol may be good enough to be quantified even after more than 10 years under −25 °C storage^32^, while oxysterols^33^ are still considerable for long-term storage at −70 °C.

### Suggestion for further studies

In the current study, we could not measure the total cholesterol, HDL, LDL, VLDL, and triglyceride in maternal blood and amniotic fluid. The measurement of these components and evaluation the relationship between cholesterol metabolic profiling and dyslipidemia would be of great help in the interpretation of the current study result. As the association between preeclampsia and dyslipidemia is not well established, more studies are needed in this issue.

In contrast to the altered metabolism of cholesterol in maternal blood, the cholesterol metabolic profiling did not show any difference in amniotic fluid. This finding is inconsistent with the result in cord blood, in which HDL and total cholesterol were lower and atherogenic incidies were increased in neonates with growth restriction^33^. As preeclampsia and fetal growth restriction are known to share the similar pathogenesis, defective placental invasion, the cholesterol metabolic profiling of cord blood will be also needed.

In recent reports, lipid-lowering agents such as pravastatin or simvastatin were reported as a therapeutic candidate for preeclampsia pathogenesis, because these medications reduced secretions of anti-angiogenic mediators such as sFlt-1^34,35^. The altered cholesterol metabolism and application of these lipid-lowering agents should be evaluated in further studies.

## Conclusion

The quantitative metabolic signatures of cholesterol suggest increased both cholesterol biosynthesis and accumulation by reverse cholesterol transport from cholesteryl esters in maternal blood (but not amniotic fluid) of women with preeclampsia.

## Data Availability

The datasets analyzed during the current study are available from the corresponding author on reasonable request.
